# Naturally acquired adaptive immunity to *Streptococcus pneumoniae* is impaired in rheumatoid arthritis patients

**DOI:** 10.1002/cti2.70012

**Published:** 2024-10-15

**Authors:** Giuseppe Ercoli, Hugh Selway‐Clarke, Dena Truijen, Milda Folkmanaite, Tate Oulton, Caitlin Norris‐Grey, Rie Nakajima, Philip Felgner, Brendan W Wren, Kevin Tetteh, Nicholas J Croucher, Maria Leandro, Geraldine Cambridge, Jeremy S Brown

**Affiliations:** ^1^ UCL Respiratory, Division of Medicine, Rayne Institute University College London London UK; ^2^ Department of Infection Biology London School of Hygiene & Tropical Medicine London UK; ^3^ Centre for Rheumatology and Bloomsbury Rheumatology Unit, Division of Medicine University College London London UK; ^4^ Vaccine Research and Development Center, Department of Physiology and Biophysics University of California Irvine Irvine CA USA; ^5^ MRC Centre for Global Infectious Disease Analysis, Department of Infectious Disease Epidemiology School of Public Health, Imperial College London London UK

**Keywords:** anti‐protein antibody, B‐cell depletion, immunosuppression treatments, rheumatoid arthritis, *Streptococcus pneumoniae*

## Abstract

**Objectives:**

Patients with rheumatoid arthritis (RA) have an increased susceptibility to infections, including those caused by *Streptococcus pneumoniae*. Why RA is associated with increased susceptibility to *S. pneumoniae* is poorly understood. This study aims to assess the effects of RA and B‐cell depletion therapy on naturally acquired antibody responses to 289 *S. pneumoniae* protein antigens using a novel protein array.

**Methods:**

IgG responses to *S. pneumoniae* were characterised in serum from RA patients and disease controls (myalgic encephalomyelitis/chronic fatigue syndrome (ME/CFS)) using whole‐cell ELISA, a flow cytometry opsonisation assay and an *S. pneumoniae* protein array. For the RA patients, results were compared before and after B‐cell depletion therapy.

**Results:**

Compared to a well‐characterised disease control group of ME/CFS patients, RA patients had reduced antibody responses to multiple *S. pneumoniae* protein antigens, with significant IgG recognition of approximately half the number of antigens along with reduced median strengths of these responses. Reduction in multiple array antigen‐specific responses also correlated with reduced IgG opsonisation of *S. pneumoniae*. Although B‐cell depletion therapy with rituximab did not reduce overall IgG recognition of *S. pneumoniae* in the RA group, it was associated with marked disruption of pre‐existing IgG repertoire to protein antigens in individual patients.

**Conclusion:**

These data show RA is associated with major disruption of naturally acquired adaptive immunity to *S. pneumoniae*, which can be assessed rapidly using a protein antigen array and is likely to contribute towards the increased incidence of pneumonia in patients with RA.

## Graphical abstract

The study highlights reductions in the level of IgG to pneumococcal proteins in sera from rheumatoid arthritis (RA) patients independent of total IgG levels, indicating one reason why RA patients have a high incidence of *Streptococcus pneumoniae* infections and pneumonia. This impairment of naturally acquired IgG to *S. pneumoniae* protein antigens was present in RA patients before B‐cell depletion treatment, although treatment with rituximab was associated with a dysregulation of the antibody responses in some patients as well.

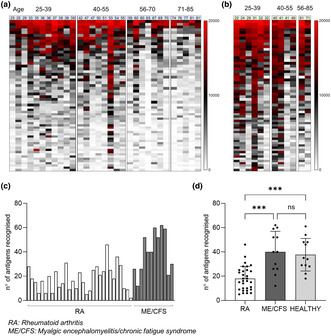

## Introduction

Rheumatoid arthritis (RA) is a chronic immune‐mediated disease in which a chronic inflammatory process leads to organ damage, predominantly in the joints but also in the lungs and the vascular system.^1^ RA patients have an increased risk of serious infections both because of the dysregulation of their immune system caused by the disease and because of treatment with immunosuppressive agents.^2^ Defects in innate and adaptive immunity contribute to this increased infection risk including reductions in TCR repertoires and capacity for clonal expansion of naïve T cells,^3^, ^4^, ^5^ and the effects of immunosuppressive drugs such as TNF‐α inhibitors, corticosteroids^2^, ^6^ and B‐cell depletion therapy (anti‐CD20, rituximab).^7^, ^8^ Pneumonia is the most prevalent serious infective complication of RA,^9^ with a greater than two‐fold increase in risk of pneumonia than the general population.^10^, ^11^, ^12^ *Streptococcus pneumoniae* (pneumococcus) is the commonest cause of pneumonia in the general population,^13^, ^14^ and immunocompromised adults have a greatly increased risk of pneumococcal infection.^15^, ^16^

Adaptive immunity to *S. pneumoniae* was previously considered to be mainly dependent on anti‐capsule antibody, and capsular antigen is used for all licenced existing *S. pneumoniae* vaccines. However, more recent data have demonstrated that recurrent episodes of nasopharyngeal colonisation with *S. pneumoniae* induce antibody response to multiple *S. pneumoniae* protein antigens,^17^, ^18^, ^19^ which provide naturally acquired protection against *S. pneumoniae* infections.^20^ This repertoire of antibody responses to *S. pneumoniae* proteins can be measured by probing arrays displaying up to 2000 protein antigens,^21^, ^22^ and this has demonstrated that human sera contain IgG to dozens of *S. pneumoniae* proteins. Each individual's repertoire of responses is distinct from others providing an antibody fingerprint for that individual. At present, there are no published data on the antibody repertoire of RA patients to *S. pneumoniae* protein antigens, and there are no data on how B‐cell depletion treatment may affect these responses. Low total IgG levels are known to increase susceptibility to respiratory infections including those caused by *S. pneumoniae*,^23^, ^24^ it is also possible in some subjects measuring total IgG within the normal range but having low levels of pathogen‐specific antibody leaving the subject at risk of specific infections. B‐cell depletion therapies are routinely used to treat an increasing range of autoimmune (including RA) and neoplastic conditions, and could specifically increase risk to *S. pneumoniae* infection by reducing naturally acquired IgG to protein antigens. Vaccines can reduce susceptibility to *S. pneumoniae*, but identifying which subjects are most at risk of infection is difficult at present.

In this study, we have used a protein array to compare IgG responses to 289 *S. pneumoniae* protein antigens in RA subjects compared to control subjects and assessed the effects of treatment with rituximab. The disease control group included well‐characterised patients with ME/CFS, who lacked clinical or serological evidence of autoimmune disease and had not been previously treated with corticosteroids or immunosuppressive agents.^25^

## Results

### Description of RA, ME/CFS and healthy control cohorts

Demographic data for the RA, ME/CFS and healthy control cohorts are shown in Table 1. The RA cohort consisted of 31 patients with a median age of 53.5 years, 32.3% of whom were male (Table 1). Data about previous and concurrent therapies were available for 24 out 31 RA patients. Most patients had previously been treated or were at the time of sampling still treated with disease‐modifying anti‐rheumatic drugs (DMARDS) with six out of 24 patients (25%) also receiving long‐term treatment with oral prednisolone (5 mg or less) (Table 1 and Supplementary table 1). The median duration of disease before the first rituximab treatment was 8 years. All RA patients have been treated with Rituximab at least once. Individual sera were collected before and after B‐cell depletion with rituximab (median 2.5 months post rituximab). Serum samples were obtained from 12 ME/CFS subjects (median age 36 years, range 22–71, none receiving immunosuppressive agents or corticosteroids) collected 6–12 months apart as controls (Table 1). Sera collected from healthy volunteers at a single time point were also included to demonstrate that ME/CFS subjects did not have reduced total and *S. pneumoniae* antigen‐specific IgG levels (Table 1, Supplementary figure 1).

**Table 1:** Demographic, disease and treatment details for the RA, ME/CFS and healthy control groups

Rheumatoid arthritis subjects (*n* = 31)	
Age (years; median, range in parentheses)	53.5 (25–81)
Sex (female/male)	21/10 (67.7%/32.3%)
Median (range) age in years of onset of RA	32.5 (2–76)
Median (range) age in years of first RTX treatment	51 (24–81)
Median (range) duration in years of RA before RTX treatment	8 (0.5–30)
Previous treatments (number of patients)	Methotrexate (24), sulfasalazine (18), hydroxychloroquine (12), gold (1), leflunomide (1), azathioprine (1), non‐rituximab biological agents (20)
Concomitant treatments (number of patients)	Prednisolone (4), methotrexate (9), sulfasalazine (5), hydroxychloroquine (6), azathioprine (1), none (8)
Rituximab treatments (number of cycles)	1 (12), 2 (2), 28 (1)
Interval between RTX treatment and post‐RTX sample (IQR months)	3.5 (1–24)
Pre‐treatment IgG (median g L^−1^, range in parentheses)	11.80 (4.51–21.92)
Post‐treatment IgG (median g L^−1^, range in parentheses)	11.46 (4.29–18.14)
Pre‐treatment IgA (median g L^−1^, range in parentheses)	2.72.(1.03–6.84)
Post‐treatment IgA (median g L^−1^, range in parentheses)	2.68 (1.39–5.06)
Pre‐treatment IgM (median g L^−1^, range in parentheses)	1.20 (0.30–2.79)
Post‐treatment IgM (median g L^−1^, range in parentheses)	0.92 (0.27–8.60)
Chronic fatigue syndrome patients (*n* = 12)	
Age (years; median, range in parentheses)	36 (22–71)
Sex (female/male)	7/5 (58.3%/41.6%)
Treatments including rituximab (number of patients)	None
First sample total IgG (median g L^−1^, range in parentheses)	10.1 (4.8–20.7)
Healthy controls (*n* = 12)	
Age (years; median, range in parentheses)	49 (22–73)
Sex (female/male)	7/5 (58.3%/41.6%)
Treatments including rituximab (number of patients)	None
Total IgG (median g L^−1^, range in parentheses)	12.70 (5.9–14.4)

### IgG recognition of *S. pneumoniae* protein antigens was markedly reduced in RA subjects

**Figure 1:**
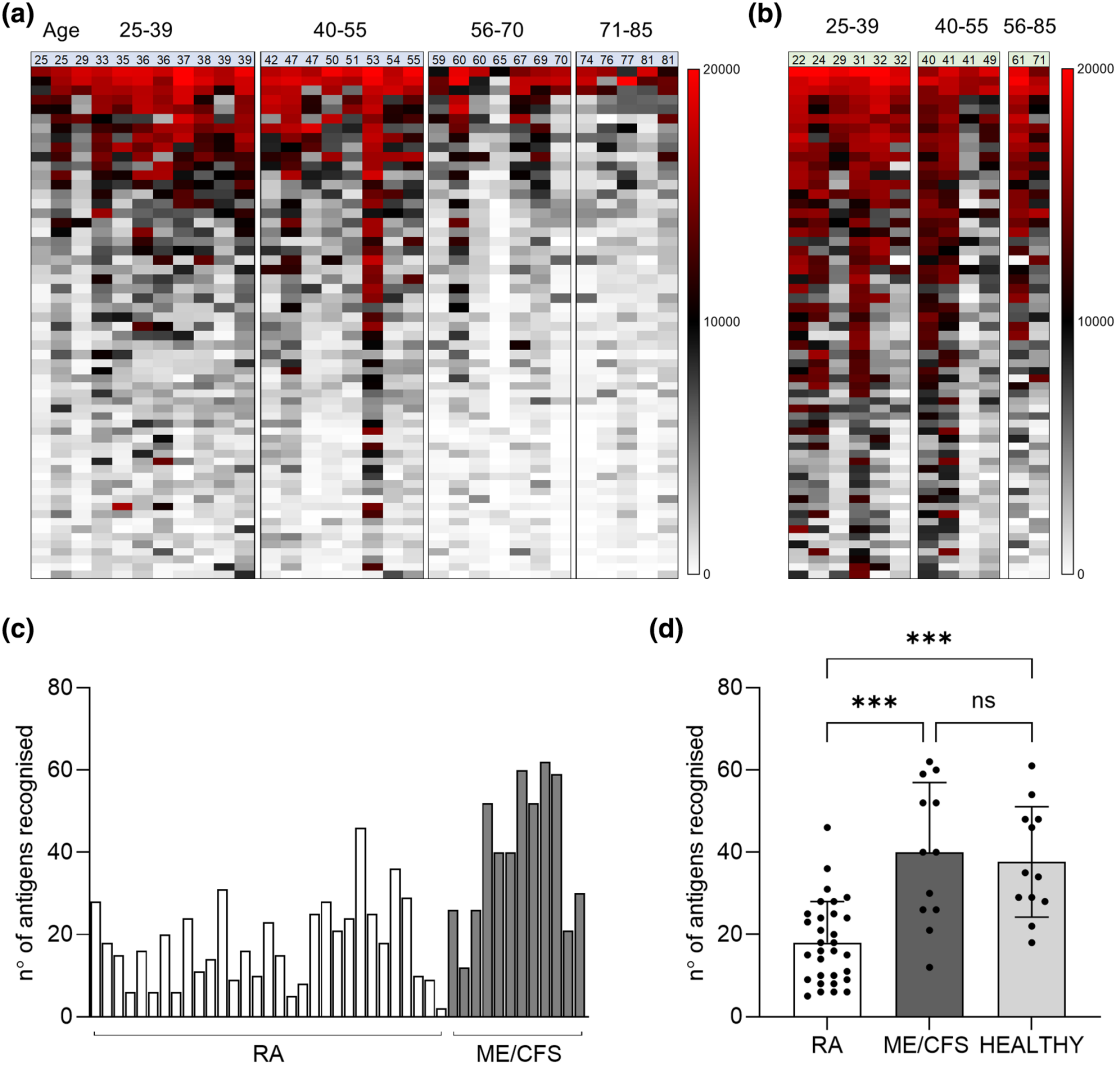
Measurement of *S. pneumoniae* antigen‐specific IgG levels using the protein array. **(a, b)** Antigen‐specific response for the top 50 recognised antigens measured by protein array for **(a)** RA subjects (pre‐rituximab therapy) and **(b)** ME/CFS patients (CFS). Data have been clustered based on patients' age (shown in the top) in four different cohorts, and ranked from most (top) to lowest (bottom) recognised antigens. Signal strength varies with colour from high (MFI = 20 000, red), medium (MFI = 10 000, black through grey) and low/absent (MFI = 0, pale grey to white). **(c)** Total number of antigens recognised by individual RA and ME/CFS control sera. Responses to a specific antigen were considered positive when the MFI measured for the sera was higher than negative control sample (naïve mouse serum) and MFI > 3000. **(d)** Overall mean number of antigens recognised for the RA and control groups. Columns indicate mean values and error bars represent standard deviations; the data were analysed using the Kruskal–Wallis test with Dunn's correction (***P < 0.001, ns not significant). ME/CFS, myalgic encephalomyelitis/chronic fatigue syndrome; RA, rheumatoid arthritis.

**Figure 2:**
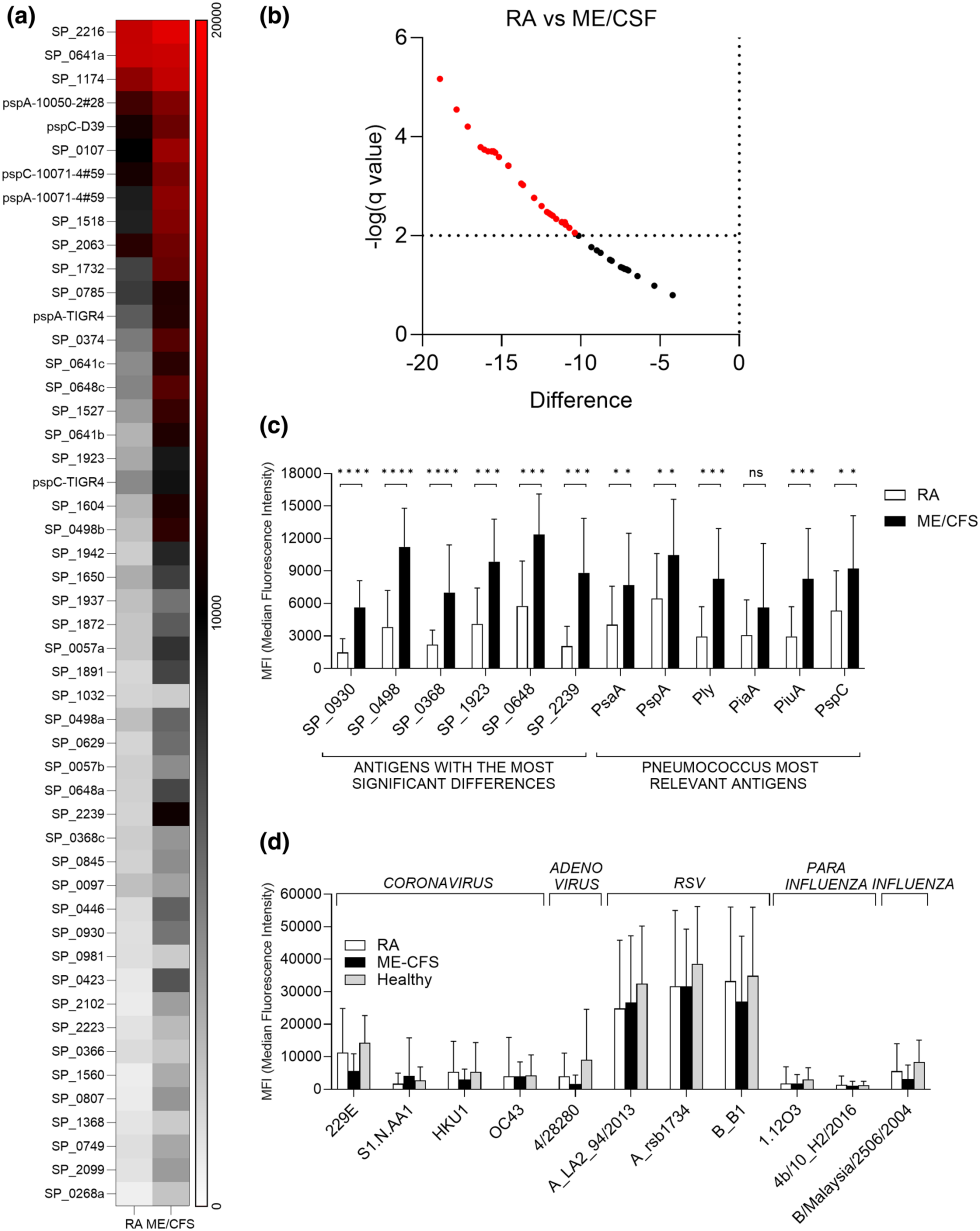
Comparison of antigen‐specific array data for RA and control subjects. **(a)** Heatmap of the mean MFI values for the top 50 recognised antigens for RA and ME/CFS cohorts ranked from most (top) to lowest (bottom) recognised. Signal strength varies with colour from high (MFI = 20 000, red), medium (MFI = 10 000, black through grey) and low/absent (MFI = 0, pale grey to white). **(b)** Volcano plot (top left quadrant) comparing results for individual antigens between RA and ME/CFS subjects. Antigens with higher signal in ME/CFS compared to RA subjects are reported and those with statistically significant differences are shown in red (dotted line indicates the threshold for statistical significance calculated using the Mann–Whitney *U*‐test with false discovery rate approach). **(c)** Mean MFI value measured by protein array for selected individual pneumococcal antigens. Error bars represent standard deviations, and the data were analysed using the unpaired Mann–Whitney *U*‐test corrected for multiple comparisons with the Benjamini, Krieger and Yakuteli method for statistical analysis (****P < 0.0001, ***P < 0.001, **P < 0.01, ns not significant). **(d)** IgG Response to human virus protein antigens was measured by protein array in RA, ME/CFS and Healthy volunteers. Response to 11 viral antigens is shown (coronaviruses, RSV, parainfluenza, adenovirus, influenza) and average MFI is shown with error bars representing standard deviations. Statistical analysis has been performed using two‐way ANOVA (none of the comparisons was statistically significant).

To provide a detailed analysis of naturally acquired adaptive immunity to *S. pneumoniae*, IgG levels were measured in sera from RA and ME/CFS control patients using a protein array containing 289 antigens, including the majority of antigens recognised in normal human serum.^20^, ^21^ Protein array measures specifically the anti‐protein IgG levels in the sera and data is not influenced by previous *S. pneumoniae* vaccination, which is dependent on capsular polysaccharide antigens. Sera from RA and control patients recognised multiple *S. pneumoniae* protein antigens (Figure 1a and b). The total number of antigens recognised by each serum varied between subjects (Figure 1c), with the mean number of *S. pneumoniae* antigens recognised by the RA cohort was 18 versus 40 for control sera (*P* < 0.001, Figure 1d) and it was not influenced by sex (Supplementary figure 1b). Measuring IgG to *S. pneumoniae* protein antigens for healthy volunteers' sera confirmed that these showed no significant difference to the results for the ME/CFS cohort (Figure 1d). The most recognised antigens were broadly conserved across most RA and control samples and were similar to those identified by Croucher *et al*. using sera from normal human subjects.^21^ However, visual inspection of the heatmap of protein array data for individual sera suggested control subjects in general had higher levels of IgG to *S. pneumoniae* protein antigens than RA patients. Mean aggregate IgG levels to *S. pneumoniae* protein antigens for sera from RA patients were lower than those for sera from controls (Figure 2a), and RA subjects had significantly weaker serum responses to multiple individual antigens compared to controls (Figure 2b and c), including for important immunodominant antigens such as PspA, PsaA, Ply, PiuA and PiaA. These results indicate RA patients have significant disruption of naturally acquired IgG‐mediated immunity to *S. pneumoniae* protein antigens. To confirm that this disruption was specific to pneumococcal protein antigens a viral protein array including a number of human virus protein antigens was also probed with the patients' sera. Results shown in Figure 2d demonstrate no significant differences in the response to different viruses between the RA and control cohorts.

### Correlations of array data to immune parameters and age

**Figure 3:**
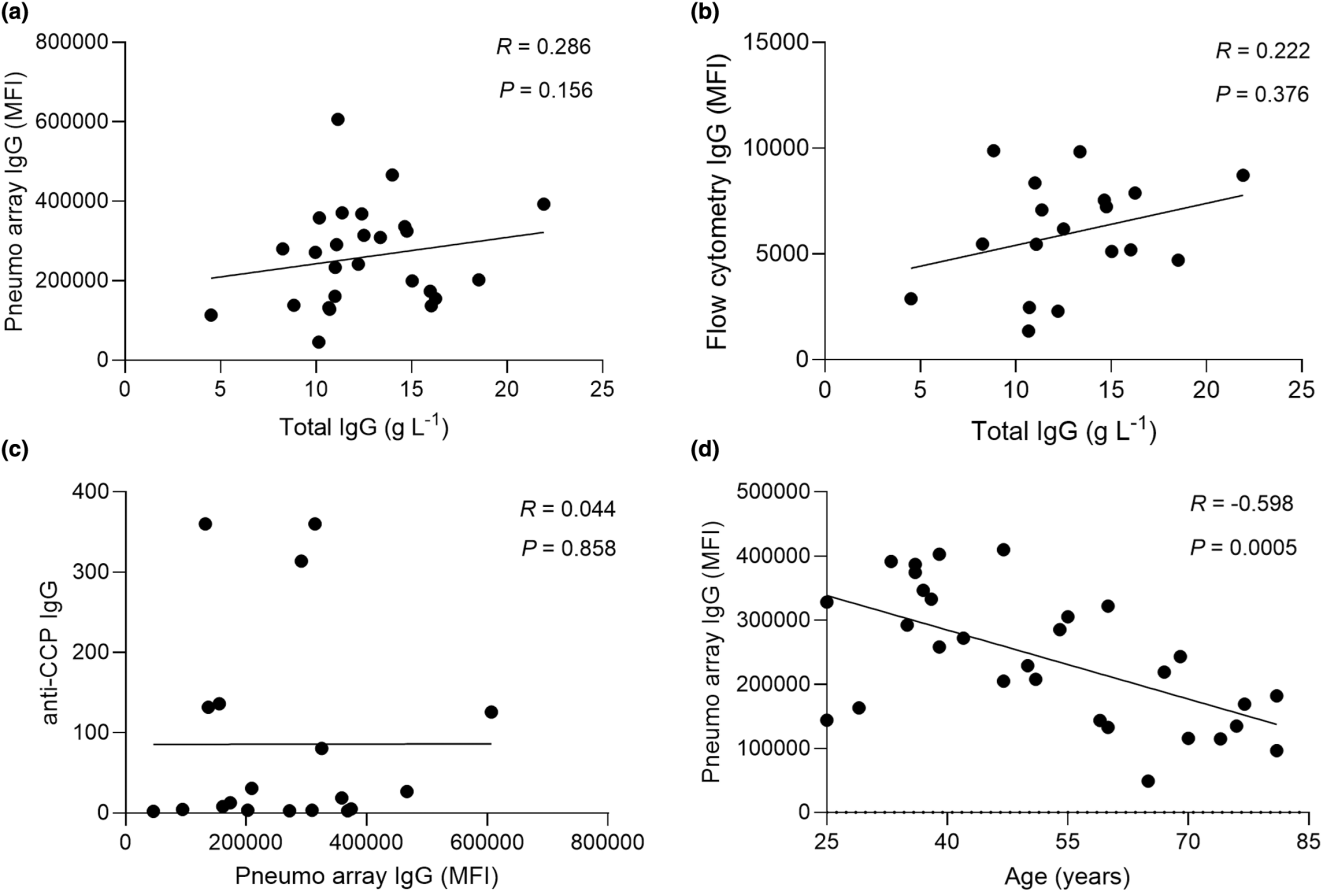
Correlation of *S. pneumoniae* protein antigen array data from RA subjects to other immune and clinical parameters. Correlation between anti‐pneumococcus IgG measured by array versus total IgG level **(a)**, anti‐pneumococcus IgG measured by flow cytometry versus total IgG level **(b)**, anti‐CCP IgG versus anti‐pneumococcus IgG level **(c)** and pneumococcus antibody levels IgG versus patients' age **(d)** are shown. Pneumo array IgG has been calculated aggregating the 50 *S. pneumoniae* protein antigens with the highest responses. Serum autoantibody levels were measured by ELISA. Each dot represents an individual subject; linear regression was calculated for all correlations and shown as a black line; *R* and *P‐*values are reported in the top right corner.

Aggregated anti‐pneumococcus IgG levels measured by protein array (Figure 3a) and serum deposition on live bacteria (Figure 3b) did not correlate with total serum IgG levels in individual subjects, demonstrating the array results do not just represent low overall serum IgG levels. In addition, the results did not show a correlation with date of sample collection, suggesting these were unlikely to be confounded by degradation of IgG responses during sample storage (Supplementary figure 2a). RA autoantibody levels (anti‐CCP) also did not correlate with aggregated anti‐pneumococcal IgG array data (Figure 3c). The non‐rituximab treatment regimens received by each patient varied markedly (Table 1 and Supplementary table 1), preventing identification of specific non‐rituximab treatments that might be associated with lower IgG levels to *S. pneumoniae* protein antigens. Equivalent correlation analysis has also been performed for the ME/CFS sera results and reported in the Supplementary figure 2b. Although marked variations occurred within an age group, overall aggregate values of anti‐*S. pneumoniae* IgG levels correlated weakly with age and were lower for both RA and control subjects aged 56 years+ (Figure 3d, Supplementary figure 2c). To account for the 20‐year difference in median age between ME/CFS and RA, the array data were reanalysed by only including the 24‐ to 55‐year‐old subjects. Results were consistent, with reduced IgG recognition of *S. pneumoniae* protein antigens in serum from RA subjects compared to controls (Supplementary figure 3).

### Array results correlated with IgG opsonisation of live *S. pneumoniae*

**Figure 4:**
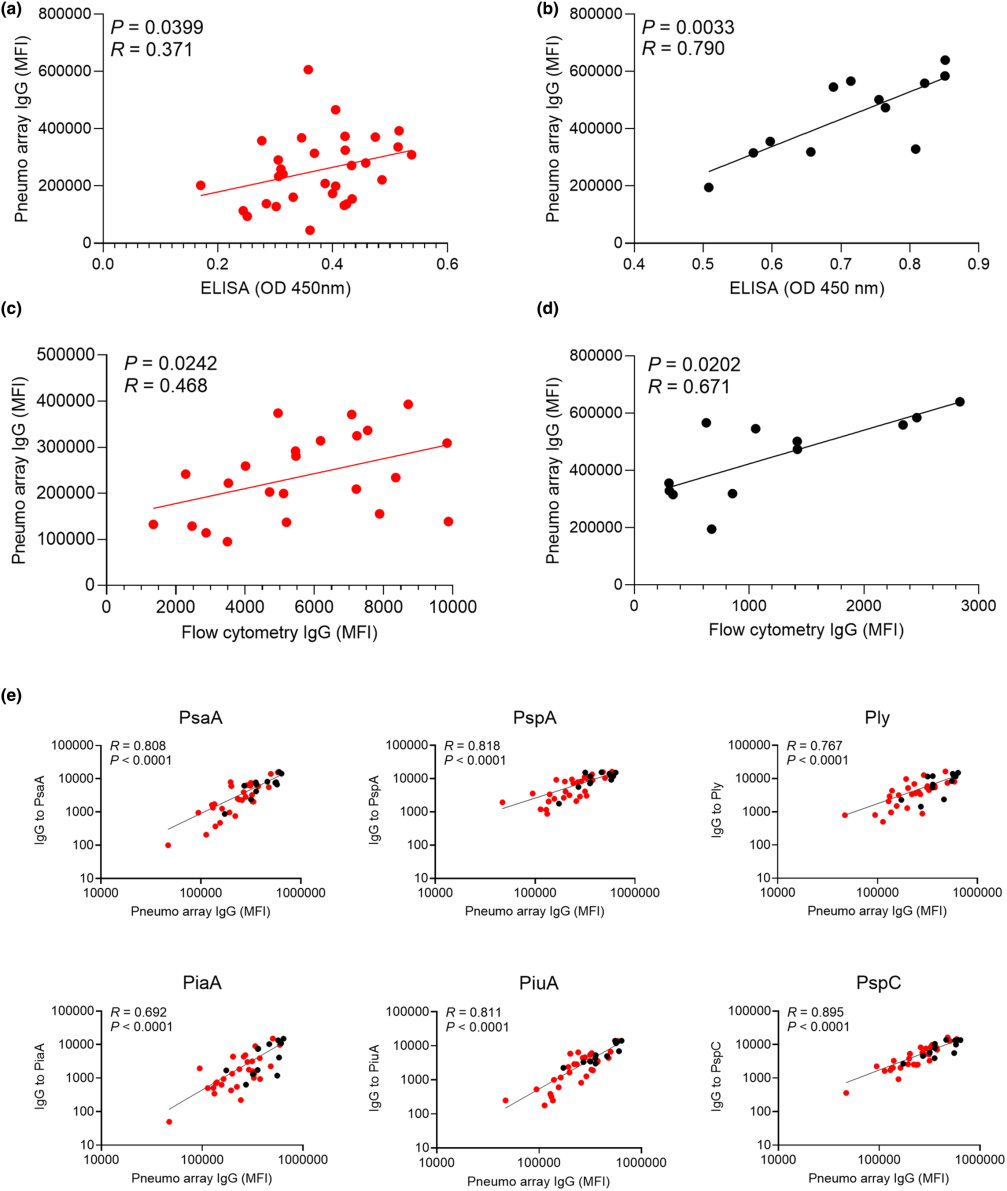
Correlations between array, whole‐cell ELISA and flow cytometry IgG opsonisation data. Pneumo array IgG was calculated by aggregating IgG levels to the 50 *S. pneumoniae* protein antigens with the highest median responses and then correlated for each individual to whole‐cell ELISA **(a, b)** and flow cytometry IgG opsonisation **(c, d)** data obtained for the TIGR4 *S. pneumoniae* strain for RA **(a, c)** and ME/CFS **(b, d)** subjects. **(e)** Correlations between the IgG levels measured using the array for six specific antigens (PsaA, PspA, Ply, PiaA, PiuA and PspC) and flow cytometry IgG opsonisation data for RA (red symbols) and ME/CFS (black symbols). Each symbol represents an individual subject; linear regression was calculated for all correlations and shown as a black line; *R* and *P‐*values are reported in the top left corner.

To link protein array data results with potential functional implications, the aggregate of IgG binding to protein antigens for individual sera was correlated with whole‐cell ELISA (measures antibody responses to all antigens, not just surface antigens) and IgG flow cytometry (measures IgG opsonisation of live *S. pneumoniae* and correlates with neutrophil phagocytosis)^20^, ^26^ obtained using the *S. pneumoniae* TIGR4 (capsular serotype 4) strain. Both the whole‐cell ELISA and flow cytometry IgG opsonisation data correlated with aggregated IgG array data for RA and control subjects (Figure 4a–d), with IgG flow cytometry data showing a stronger correlation for RA subjects than the whole‐cell ELISA results.

To identify specific antigens where array IgG responses were a marker for improved opsonisation, array data for selected antigens with high levels of IgG binding were correlated with the flow cytometry IgG opsonisation results in RA and control sera. Opsonisation of *S. pneumoniae* with IgG correlated with array IgG levels to PsaA, PspA, Ply, PiaA, PiuA and PspC (Figure 4e), most of which had significantly reduced responses in RA sera compared to controls (Figure 2c). Weaker correlations were observed for SP_2216 and SP_0641, antigens for which there were very high levels of specific IgG in most sera (Supplementary figure 4). There was no correlation between IgG opsonisation and specific IgG to SP_1411, an antigen with low levels of IgG recognition in most serum samples (Supplementary figure 4). Overall, these data support that variations between subjects in IgG recognition of specific antigens were likely to have functional consequences for immune recognition and clearance of *S. pneumoniae*.

### The effects of rituximab treatment on IgG recognition of *S. pneumoniae*

**Figure 5:**
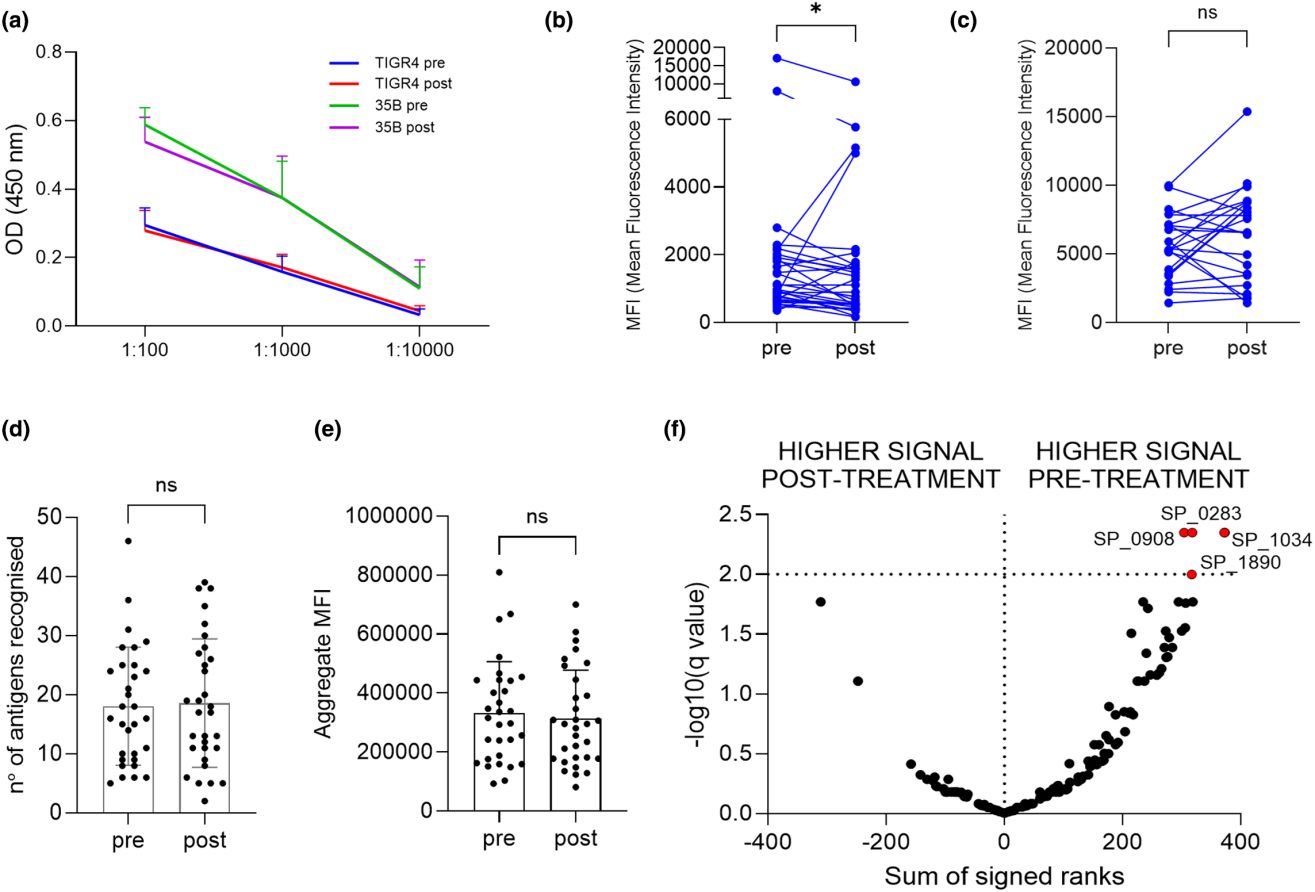
Overall effects of rituximab treatment on IgG recognition of *S. pneumoniae* in RA subjects. **(a)** Whole‐cell ELISA IgG recognition of *S. pneumoniae* TIGR4 and 35B (not included in Pneumovax vaccine) strains in serum from RA subjects pre‐ and post‐rituximab treatment. **(b, c)** Flow cytometry IgG opsonisation of *S. pneumoniae* **(b)** TIGR4, and **(c)** 35B strains in serum from RA subjects pre‐ and post‐rituximab treatment. Differences between pre‐ and post‐rituximab treatment samples were analysed using the Wilcoxon signed‐rank test. *P‐value < 0.05; ns, not significant. **(d, e)** Array data showing **(d)** total number of antigens recognised and **(e)** mean aggregate MFI value for top 50 antigens in pre‐ and post‐rituximab treatment samples. Symbols represent results for individual subjects, bars correspond to mean values for the cohort, error bars indicate standard deviation. Data were analysed using the Mann–Whitney *U*‐test and showed no statistically significant differences between pre‐ versus post‐rituximab treatment sera. **(f)** Volcano plot comparing results for individual antigens between pre‐ versus post‐rituximab treatment samples for RA subjects. Right side of the plot represents antigens with higher results in pre‐rituximab sera compared to post‐rituximab, antigens indicated with the red dots showed statistically significant changes (the plot has been calculated on the top‐ranked 170 antigens applying the Wilcoxon matched‐pairs rank test to each row and the two‐stage linear step‐up procedure of Benjamini, Krieger and Yekutieli).

Rituximab therapy of RA patients had a small effect on total serum IgG, with a mean decrease of 1.13 g L^−1^ (SD 1.94, *P* = 0.0124) in post‐rituximab sera. To investigate whether this was associated with changes in IgG to *S. pneumoniae*, whole‐cell ELISA and flow cytometry assay data were compared before and after rituximab treatment for the RA patients. Importantly, these assays showed no significant differences in baseline and the 6‐month sera samples from control patients (Supplementary figure 5). In RA patients, there were also no detectable effects of B‐cell depletion treatment on whole‐cell IgG ELISA for two *S. pneumoniae* strains (serotype 4 and 35B) (Figure 5a). The flow cytometry assays demonstrated a fall in IgG opsonisation for the serotype 4 but not the 35B strain in the post‐rituximab samples (Figure 5b and c). When the array data were compared between pre‐ and post‐rituximab samples from RA patients, there were no overall differences in the total number of antigens recognised and aggregate total IgG MFI to protein antigens (Figure 5d and e). Analysis of array data for the top 170 ranked antigens showed a significant fall in post‐rituximab sera for four proteins: SP_0283, SP_0908, SP_1034 and SP_1890 (Figure 5f).

**Figure 6:**
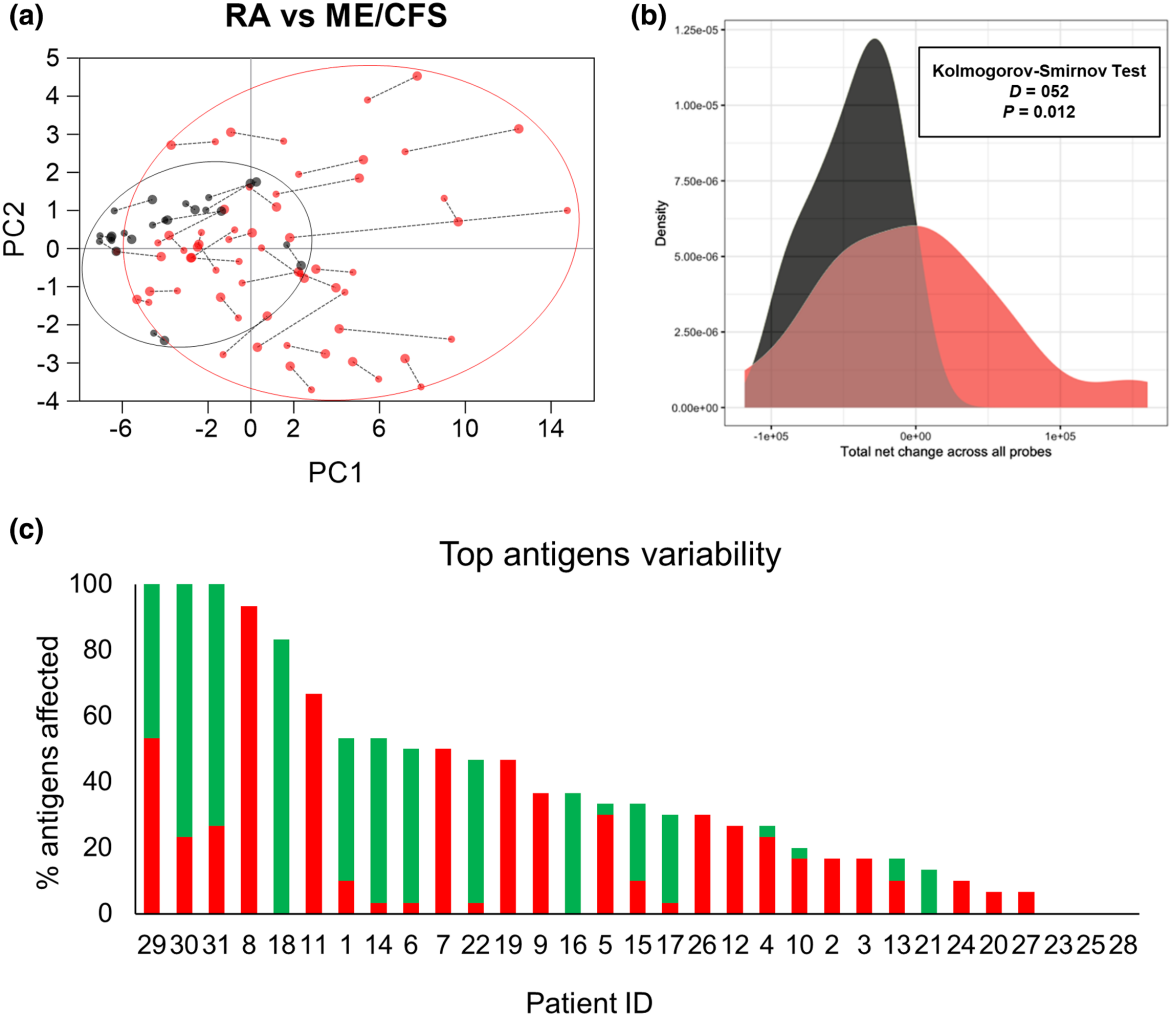
Variability of anti‐protein response after B‐cell depletion in RA patients. **(a)** PCA analysis of protein array data for the top 30 most recognised antigens in pre‐ versus post‐rituximab treatment sera from RA patients (red symbols), and for samples from each ME/CFS (control) subject obtained 6 months apart (black symbols). The change in array data between the two samples from an individual subject is proportional to the length of the dotted line connecting the first (small dot) to the second (big dot) sample. **(b)** Kolmogorov distribution has been calculated to test whether there was a difference in the change between timepoints, the sum of differences across all probes was calculated for each patient for both RA (Red) and ME/CFS (black) cohorts. Statistical significance of the difference was calculated using the Kolmogorov–Smirnov test. **(c)** Percentage of antigen‐specific response showing significant differences (higher or lower than average change in ME/CFS cohort) (Supplementary table 2) between pre‐ and post‐rituximab sera. Data are reported for the top‐ranked 30 antigens with the green portion of each column representing antigens with increases in IgG in post‐rituximab samples and red with decreases in IgG in post‐rituximab samples. Subjects were ranked from right to left according to total number of antigens with significant differences between pre‐ and post‐rituximab treatment sera. Patients ID numbers are displayed on the *x*‐axis.

Overall, these data demonstrate little effect from rituximab treatment on IgG responses to *S. pneumoniae* when assessed using all RA patients. However, IgG opsonisation of *S. pneumoniae* for some RA patients showed a fall in post‐rituximab samples, with others showing an increase (Figure 5b and c), indicating that for some individuals there may have been significant disruption of pre‐existing IgG to *S. pneumoniae* in post‐rituximab sera. Principal component analysis (PCA) of pre‐ and post‐rituximab samples provided support for this; the 30 top recognised antigens have been identified by plotting the median response across the population (Supplementary figure 6a) and used to calculate the PCA. Several RA subjects showed considerable shifts in their IgG responses to protein antigens which contrasted to our sequential data from controls, and previously published longitudinal data for normal subjects (Figure 6a and b).^21^, ^27^ T‐SNE and UMAP analysis have been also performed to confirm the findings and reported in the Supplementary figure 6b and c.

Furthermore, when changes in IgG recognition for the top 30 antigens were analysed for each RA subject pre‐ and post‐rituximab treatment, 24 of 31 subjects showed significant changes in IgG recognition of five or more antigens (Figure 6c, Supplementary figure 7). The direction of change varied with a relatively even distribution between patients showing increased, decreased or mixed changes to multiple antigens. These data indicate that in some RA subjects, rituximab therapy was associated with significant disruption of pre‐existing antigen‐specific IgG that could potentially either weaken or strengthen adaptive immunity to *S. pneumoniae*.

## Discussion

Rheumatoid arthritis patients have a fourfold increased risk of developing pneumonia caused by *S. pneumoniae*,^15^, ^16^ but the underlying immune defects causing this increased incidence of *S. pneumoniae* infections have been poorly explored. Using a protein antigen array, we have characterised in detail systemic IgG responses to multiple *S. pneumoniae* protein antigens in sera from RA subjects. The results were compared to a patient control group of ME/CFS patients, selected as a disease whose onset is commonly linked to viral or bacterial infections^28^ but with limited evidence for autoimmunity or major immune disruption. The main findings were (i) sera from RA subjects contained IgG to fewer *S. pneumoniae* protein antigens and had reduced strength of responses to specific antigens; (ii) array data correlated with IgG opsonisation of *S. pneumoniae* in sera, which correlates with neutrophil uptake and therefore immunity to *S. pneumoniae* ^20^, ^26^ and (iii) rituximab therapy did not have major additional effects on IgG recognition of *S. pneumoniae* protein antigens in this cohort of RA patients. The latter is perhaps the most surprising finding, but it is in line with our previously published data on anti‐CCP antibody levels in RA patients.^29^ These data identify one mechanism that increases susceptibility to *S. pneumoniae* in RA patients and demonstrate a potential use for protein arrays for identifying patients at high risk of serious infection.

Despite the previous emphasis on anti‐capsular antibody as the main mechanism of adaptive immunity to *S. pneumoniae*, there is now considerable human and animal experimental data defining the important mechanisms for naturally acquired (as opposed to vaccine‐induced) immunity to *S. pneumoniae*.^20^, ^27^, ^30^, ^31^, ^32^ Humans develop antibody to multiple protein antigens in response to nasopharyngeal colonisation events, which results in IgG‐mediated opsonisation of *S. pneumoniae* that protects against systemic infection and pneumonia.^20^, ^21^, ^22^, ^33^ Hence, our finding that IgG recognition of protein antigens is impaired in RA subjects and correlates with reduced opsonisation of live *S. pneumoniae* with IgG is likely to be clinically relevant and increase susceptibility to *S. pneumoniae*. RA patients are eligible for vaccination against pneumococcus using capsular antigen vaccines but ME/CFS or healthy controls are not, and this would confound comparing results of anti‐capsular IgG responses between these groups. Other mechanisms are also likely to reduce immunity to *S. pneumoniae* subjects, including additional disease or drug effects on innate and/or cellular immunity, and background RA‐associated lung disease. Teasing out the relative contributions of naturally acquired antibody and other mechanisms for RA‐related susceptibility to *S. pneumoniae* will require more detailed studies of pathogen‐specific immunity combined with prospective studies of infectious complications in RA patients.

Why RA patients have reduced levels of naturally acquired IgG to protein antigens will also require further investigation. The results seem to be specific for *S. pneumoniae* antigens as there were no significant differences between groups in anti‐viral IgG responses, and were not driven by differences in total IgG levels between subjects. Many of the treatments of RA, including methotrexate, systemic corticosteroids and biological therapies can reduce antibody responses to vaccines (including *S. pneumoniae* vaccines).^34^, ^35^, ^36^, ^37^, ^38^ These drugs are therefore likely to impair the boosting of naturally acquired antibody to protein antigens by repeated *S. pneumoniae* nasopharyngeal colonisation events and potentially established memory responses. Direct disease effects of RA on immune function could also potentially affect adaptive immunity to *S. pneumoniae* protein antigens. However, the maintained IgG responses to several respiratory viral antigens and the lack of correlation of *S. pneumoniae* IgG array results to auto‐antibody levels both do not support a direct effect of RA on adaptive immunity to *S. pneumoniae*. Differentiating between the effects of different immunosuppressive agents (including those such as systemic steroids and anti‐TNF alpha therapies that are known to reduce vaccine responses) and RA on immunity to *S. pneumoniae* will need careful serial measurements of serum IgG to *S. pneumoniae* protein antigens from RA subjects over the disease course and after starting new therapies.

A significant limitation of this study was the cohort's heterogeneity, compounded by the relatively small sample size and marked variations in the treatments received by the patients. This prevented identifying which medications were associated with reduced IgG to *S. pneumoniae* protein antigens. Additionally, the lack of clinical data about *S. pneumoniae* infections and carriage events limited our ability to capture dynamic changes in response to pneumococcus over time. Expanding our focus beyond humoral responses to include cellular immunity would offer a more comprehensive understanding of the immune response in RA patients. However, measuring T‐cell responses to *S. pneumoniae* in human samples would require obtaining blood lymphocytes and also remains technically challenging.

An interesting observation from our data was the relative lack of effect of rituximab therapy on *S. pneumoniae*‐specific IgG, suggesting that rituximab does not have a major additional effect on susceptibility to *S. pneumoniae* in RA subjects. This perhaps reflects the lack of rituximab effects on plasma cells that do not express CD20. However, for some individuals, rituximab was associated with considerable change within their IgG repertoire to *S. pneumoniae* protein antigens. The antibody repertoire is specific for each person and usually remains stable over time even on re‐exposure to *S. pneumoniae*,^22^ and the changes seen in some post‐rituximab samples are unusual and suggest large changes in the relative frequencies of individual B‐cell clones. The post‐rituximab effects on the IgG repertoire to *S. pneumoniae* protein antigens increased in some subjects and decreased in others, and the consequences for protection against infection are also likely to vary.

An important question is whether the reduced antibody to *S. pneumoniae* protein antigens in RA subjects reflects a wider impairment of antibody recognition for other pathogens or is a specific immune defect for *S. pneumoniae*. In addition, whether other patients with autoimmune disease and/or receiving immunosuppressive therapies also have reduced antibody to *S. pneumoniae* protein antigens needs investigating. Existing methods of identifying subjects at increased risk of infections because of disease or treatment‐related immune defects are largely restricted to measuring white cell subsets and overall immunoglobulin levels. As a consequence, we are unable to identify which patients are most at risk of specific infections. A protein array could be one tool to answer this question, allowing the rapid measurement of IgG responses to selected antigens from multiple pathogens to characterise an individual's risk of infection for specific pathogens.

In summary, we have used a protein array to demonstrate that RA subjects have reduced levels of naturally acquired serum IgG to *S. pneumoniae* protein antigens, identifying an important mechanism suggesting why RA subjects have an increased susceptibility to *S. pneumoniae*. This defect in adaptive immunity to *S. pneumoniae* could be improved by vaccination with vaccines containing capsular antigens, but under‐vaccination of RA patients remains a significant issue with under 50% of patients having had full vaccination against *S. pneumoniae*.^39^, ^40^ Our data emphasise the importance of encouraging all patients with RA to be vaccinated against *S. pneumoniae*, and provide a starting point for future detailed analyses of disease effects on immunity to specific bacterial pathogens.

## Methods

### RA and ME/CFS patients and serum samples

Sera for this study were obtained from patients with RA (*n* = 31) treated at UCLH participating in an observational prospective study (REC 08/H0715/18). Patients with active RA (DAS28 ≥ 5.1) fulfilling revised ACR/EULAR diagnostic criteria, treated with Rituximab, and with serum samples available before and after Rituximab treatment were included in the study. Sera samples were collected before the Rituximab treatment (one sample) and at least 1 month after (3 or 4 samples). Control serum samples were collected 6–12 months apart from 12 patients diagnosed with ME/CFS (fulfilling consensus criteria) selected from the cohort described in Mensah *et al*.^25^, ^41^ The study was approved by the NRES Committee London–City Road and Hampstead Research Ethics Committee (REC reference: 14/LO/0388). Samples from healthy volunteers (*n* = 12) have been also used in this study (UCL Research Ethics Committee 3078/001). Total IgG, IgM and IgA serum levels for the RA cohort were measured at the UCLH Central Lab. Isotypes of anti‐cyclic citrullinated peptide (anti‐CCP) antibody levels (IgG, IgA and IgM) were measured by ELISA as previously described.^42^ For the ME/CFS and Healthy cohorts total IgG levels were measured by ELISA (BMS2091, Thermo Fisher Scientific, Hemel Hempstead, UK).

### Whole‐cell ELISA, flow cytometry IgG and IgM binding assays

Global antibody recognition of *S. pneumoniae* was assessed using previously described whole‐cell ELISA and flow cytometry assays^20^, ^31^ using the TIGR4 (serotype 4) and a capsular‐switched TIGR4 strain expressing a serotype 35B capsule (not present in Pneumovax).^43^, ^44^ Briefly, for whole‐cell ELISA assays 50 μL/well of *S. pneumoniae* at an OD_600_ of 0.4 in PBS was added to microtiter plates, and incubated overnight at room temperature, before fixation in 4% formaldehyde for 10 min. Plates were washed and incubated with a 1:100 dilution of human serum for 1 h at 37°C, using HRP‐conjugated goat anti‐human IgG (ab7499, abCam, Cambridge, UK) for detection. For flow cytometry antibody binding assays, 1 × 10^6^ live *S. pneumoniae* CFU were incubated for 30 min at 37°C with 10% human serum followed by the addition of fluorescently labelled anti‐human IgG (410711, BioLegend, San Diego, CA) before flow cytometry using a BD FACS Verse instrument.

### IgG recognition of *S. pneumoniae* protein antigens measured using an array

A *S. pneumoniae* 289 protein array was constructed using *in vitro* cell‐free expression of selected antigens as previously described.^27^, ^31^ The selected proteins were highly conserved amongst > 600 *S. pneumoniae* strains^21^ and included all the conserved proteins recognised by IgG in human sera obtained from healthy adults.^21^ Arrays were probed with serum samples diluted 1:50 in blocking buffer (Maine Manufacturing, Sanford, ME) supplemented with *E. coli* lysate. Images were acquired and analysed using an ArrayCAM® Imaging System from Grace Bio‐Labs.^45^, ^46^ After subtraction of background, antibody responses to each antigen were ranked based on the average strength of the response on the whole dataset (highest signal > lowest signal) and presented as heatmaps of results for individual serum samples and aggregate levels for RA subjects versus controls (calculated by adding up the level of signal measured against top 50 or all the antigens). The number of positive antigens for each sample was calculated by defining a minimum threshold for each protein in the array based on the results obtained for negative control serum (naïve mouse). An antigen was considered positive if the median fluorescence measured was at least two standard deviations greater than the results for the negative control samples and above a threshold MFI of 3000. To assess significant changes in responses to single antigens in the same subjects, the average difference in MFI between time zero and 6 months samples was calculated for each antigen in ME/CFS sera (Supplementary table 2). This value added to two standard deviations was the threshold set to identify antigens with significant variations between pre‐ and post‐rituximab treatment samples for RA patients (Supplementary figure 7). IgG responses to respiratory viral antigens were measured in baseline sera from RA subjects, ME/CFS and healthy volunteers' sera using a COVAM array (Sinoimmune, SinoBiological, China) as described for the *S. pneumoniae* array. Data were not presented for IgG responses to viral antigens with either very low antibody levels or when the time of serum collection for some subjects preceded when the relevant viral strain was circulating (e.g. SARS‐Cov, SARS‐Cov2, MERS, some influenza strains).

### Statistical analysis

Using previous protein array data from mouse and human sera a sample size of 11 was powered to identify a 50 + % difference in mean number of antigens recognised and mean aggregate MFI between the RA versus ME/CFS cohorts with 80% power and 0.05 probability.

Statistical analyses were conducted using Prism 9 (Graph Pad, USA). Comparisons of data from RA, ME/CFS and Healthy volunteer cohorts have been performed either using the Kruskal–Wallis test with Dunn's correction (one variable, comparison of the three groups), the unpaired Mann–Whitney *U*‐test corrected with the Benjamini, Krieger and Yakuteli (multiple variables, comparisons of two groups) or ordinary two‐way ANOVA with Tukey's correction (multiple variables, comparisons of the three groups). The non‐parametric tests have been used for the pneumo protein array dataset (non‐normally distributed) while the parametric two‐way ANOVA has been used for the viral antigens array. Comparisons between pre‐ and post‐rituximab samples used paired multiple *t*‐tests (Wilcoxon). Correlations were analysed using non‐parametric Spearman correlations. For histograms, data are presented as means, and error bars represent standard deviations. Array data were analysed using standard dimensionality‐reduction method PCA using Prism 9 and t‐Distributed Stochastic Neighbor Embedding (t‐SNE) via the R package Rtsne^47^ with a perplexity value of 6 to visualise IgG responses across patients and time points. The analysis was rerun with a perplexity value of 5 and further rerun with Uniform Manifold Approximation and Projection (UMAP),^48^ R package UMAP, to visually confirm that the results were consistent across methods.

## Author contributions

**Giuseppe Ercoli:** Conceptualization; data curation; formal analysis; investigation; methodology; validation; visualization; writing – original draft; writing – review and editing. **Hugh Selway‐Clarke:** Conceptualization; formal analysis; visualization. **Dena Truijen:** Formal analysis; investigation; visualization. **Milda Folkmanaite:** Formal analysis; investigation; visualization. **Tate Oulton:** Formal analysis; visualization. **Caitlin Norris‐Grey:** Investigation; methodology. **Rie Nakajima:** Investigation; methodology. **Philip Felgner:** Resources. **Brendan W Wren:** Funding acquisition; resources. **Kevin Tetteh:** Resources. **Nicholas J Croucher:** Data curation; formal analysis; visualization. **Maria Leandro:** Conceptualization; data curation; writing – review and editing. **Geraldine Cambridge:** Conceptualization; data curation; formal analysis; investigation; methodology; supervision; writing – review and editing. **Jeremy S Brown:** Conceptualization; data curation; funding acquisition; project administration; supervision; writing – review and editing.

## Conflict of interest

The authors declare no conflict of interest.

## Supporting information


Supplementary table 1
Supplementary table 2
Supplementary figure 1
Supplementary figure 2
Supplementary figure 3
Supplementary figure 4
Supplementary figure 5
Supplementary figure 6
Supplementary figure 7

## Data Availability

The data supporting this study's findings are available from the corresponding author upon reasonable request.
