# Supplementary material for: Naturally acquired adaptive immunity to Streptococcus pneumoniae is impaired in rheumatoid arthritis patients
Source: Clin Transl Immunology. 2024 Oct 15;13(10):e70012. doi: 10.1002/cti2.70012 (PMC11480415; doi:10.1002/cti2.70012)
Supplement: Supplementary file 1 — Supplementary table 1 Supplementary table 2 Supplementary figure 1 Supplementary figure 2 Supplementary figure 3 Supplementary figure 4 Supplementary figure 5 Supplementary figure 6 Supplementary figure 7 [file CTI2-13-e70012-s001.pdf]

Supplementary File

Naturally-acquired adaptive immunity to *Streptococcus pneumoniae* is impaired in rheumatoid arthritis patients

Giuseppe Ercoli[a#], Hugh Selway-Clarke[a], Dena Truijen[a], Milda Folkmanaite[a], Tate Oulton[b], Caitlin Norris-Grey[c], Rie Nakajima[d], Philip Felgner[d], Brendan W Wren[b], Kevin Tetteh[b], Nicholas J Croucher [e], Maria Leandro[c], Geraldine Cambridge[c] and Jeremy S Brown[a]

[a] UCL Respiratory, Division of Medicine, University College London, Rayne Institute, London WC1E 6JF, United Kingdom.

[b] Department of Infection Biology, London School of Hygiene & Tropical Medicine, London, WC1E 7HT, UK.

[c] Centre for Rheumatology and Bloomsbury Rheumatology Unit, Division of Medicine, University College London, London, UK.

[d] Vaccine Research and Development Center, Department of Physiology and Biophysics, University of California Irvine, Irvine, California, USA.

[e] MRC Centre for Global Infectious Disease Analysis, Department of Infectious Disease Epidemiology, School of Public Health, Imperial College London, London, W12 0BZ, UK.

[#]**Correspondence:**

Mailing address:

UCL Respiratory, University College London,

Rayne Institute, 5 University Street, London WC1E 6JF, United Kingdom.

Phone : 44 20 7679 6008

E-mail : g.ercoli@ucl.ac.uk

Supplementary table 1: Previous and concomitant therapies administered to the RA cohort

	PREVIOUS THERAPY										CONCOMITANT THERAPY
	Traditional DMARDs					BIOLOGICS (TNF and IL inhibitors)					DMARDS, BIO AND STEROIDS
ID	*MTX*	*SSZ*	*HCQ*	*LEFLU*	*AZATHIO*	*ADA*	*ETANA*	*CERTOLIZ*	*INFLIXI*	*TOCILIZ*	
1	■	■					■				None
2	■	■				■					SSZ, PRD
3	■	■	■						■		MTX,SSZ,HCQ
4	■	■				■					None
5	■		■			■	■				MTX, HCQ, PRD
6	■	■				■	■				None
7	■	■	■								HXQ
8	■	■	■								MTX,SSZ,HCQ
10	■	■					■				MTX, PRD
13	■	■	■								MTX,SSZ,HCQ
14	■	■		■			■				None
15	■	■	■	■		■	■				HXQ, PRD
16	■	■	■				■			■	None
17	■	■	■			■	■				MTX
18	■						■		■		MTX
20	■	■	■			■					MTX,HCQ,SSZ
21	■					■					None
22	■	■				■	■				none
23	■	■					■	■			none
24	■		■			■	■				MTX, HCQ
27	■	■	■				■				None
28	■	■	■								SSZ, PRD
30	■	■				■					PRD
31	■	■	■		■	■	■				AZATHIO

Legend

AZATHIO azathioprine (Imuran®)
HCQ Hydroxychloroquine (Plaquenil ®)
LEFLU Leflunomide (Arava®)
MTX Methotrexate (Rheumatrex®, Trexall®)
SSZ Sulfasalazine (Azulfidine®)

ADA adalimumab (Humira ®)
CERTOLIZ Certolizumab (Cimzia®)
ETANA etanercept (Enbrel®)
INFLIXI infliximab (Remicade®)
TOCILIZ Tocilizumab (Actemra®)

PRD Prednisolone

Coloured boxes indicate a specific drug has been used for the patient. Info for patients 9, 11, 12, 19, 25, 26 and 29 were not available.

Supplementary table 2 Antigen response variability

Antigen	Average MFI (% change)	SD (% change)
SP_2216	7.34	14.59
SP_0641a	4.75	7.94
SP_1174	5.94	9.07
pspA-10050-2#28	1.29	26.32
pspC-D39	10.41	20.98
SP_0107	2.84	27.47
pspC-10071-4#59	8.00	27.93
pspA-10071-4#59	12.41	26.53
SP_1518	0.30	29.55
SP_2063	5.56	15.11
SP_1732	0.63	29.64
SP_0785	3.93	33.74
pspA-TIGR4	5.76	27.09
SP_0374	8.96	20.42
SP_0641c	6.84	25.93
SP_0648c	14.97	25.86
SP_1527	8.31	32.13
SP_0641b	15.69	24.49
SP_1923	13.35	16.95
pspC-TIGR4	17.35	16.06
SP_1604	10.04	33.41
SP_0498b	1.46	45.40
SP_1942	7.36	19.71
SP_1650	10.86	34.76
SP_1937	18.03	23.51
SP_1872	6.15	27.91
SP_0057a	9.84	26.98
SP_1891	9.71	33.80
SP_1032	16.19	17.70
SP_0498a	15.13	32.04

Variability of IgG response for individual pneumococcal antigens is reported as the average percentage change measured by protein array in sera samples from ME/CFS patients 6 months apart. Average and standard deviation have been calculated in a cohort of 12 patients for the 30 most recognised antigens.

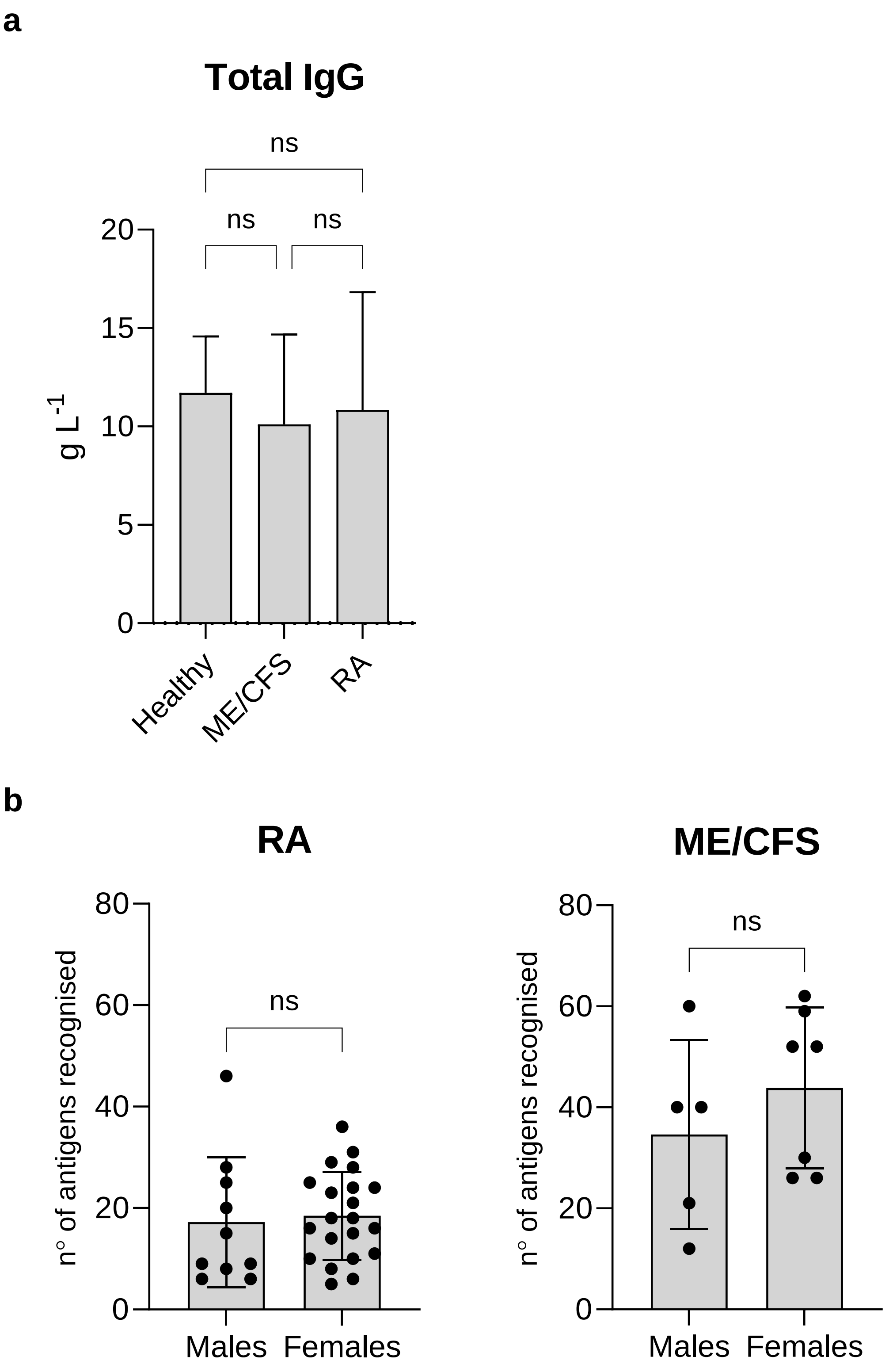

Supplementary figure 1 Total IgG level measured by ELISA. **(a)** Healthy volunteers, ME/CFS control cohort and RA patients' sera samples have been tested for total level of IgG. Mean value are reported for each cohort and error bars represent standard deviations. The data were analysed using unpaired Kruskal-Wallis test with Dunn's correction for multiple comparisons for statistical analysis (ns, not significant). **(b)** Sex dependent response to pneumococcus measured by protein array as overall mean number of antigens recognised in RA and ME/CFS cohorts. Columns indicate mean values and error bars represent standard deviations; the data were analysed using a Mann-Whitney *U*-test (ns not significant)

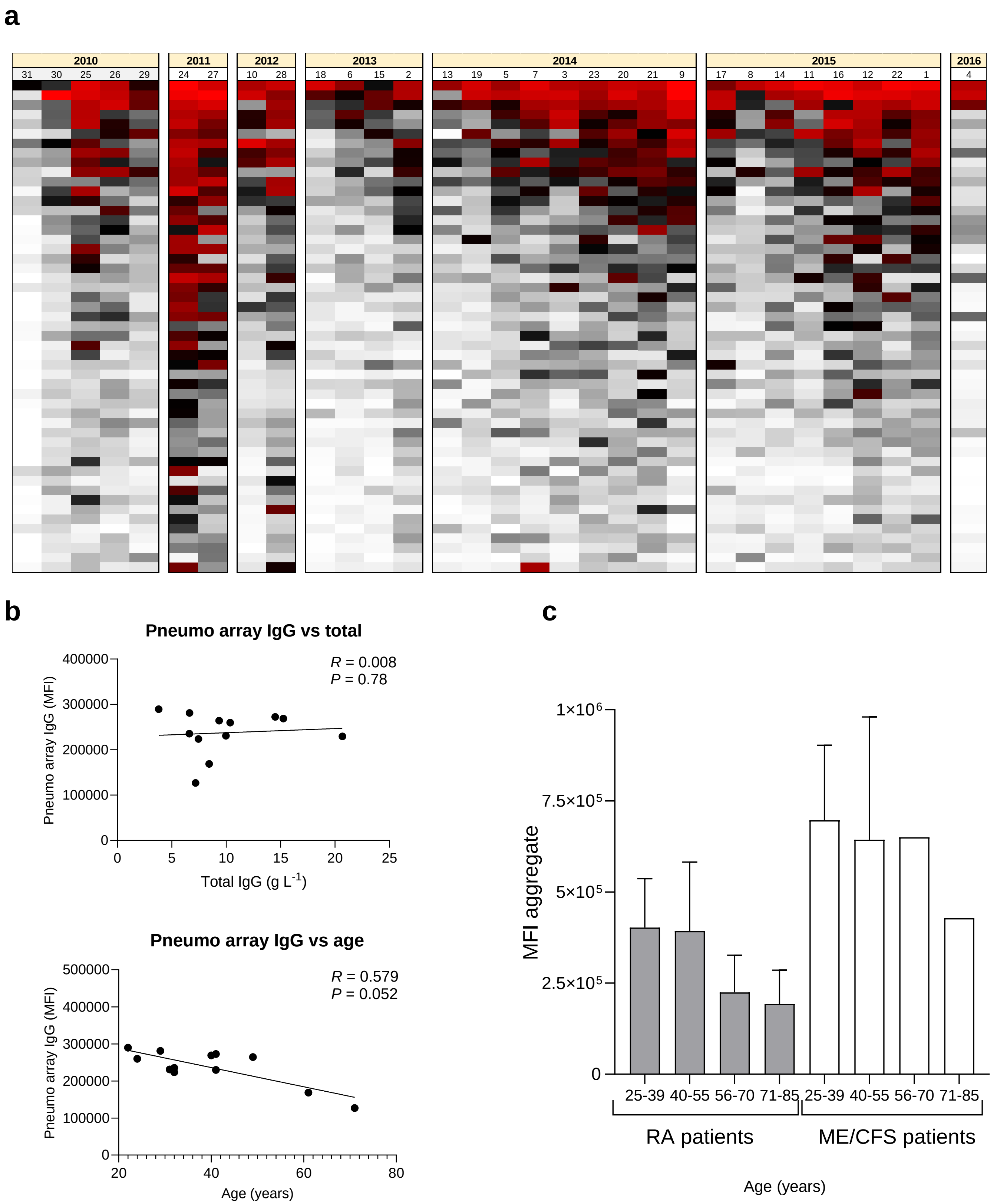

Supplementary figure 2 Time of collection and patients' age influence on array data. **(a)** Patients have been grouped based on their age at the time of sampling. Year of serum collection is reported for each patient in association with protein array data (top 50 ranked antigens) to show that reactivity levels are not affected by storage. Antigens are ranked from most recognised (top) to lowest (bottom) and signal strength varies from high (MFI=20000, red), medium (MFI=10000, black) and low (MFI=0, white). **(b)** Correlation between anti-pneumococcus IgG vs total IgG level and anti-pneumococcus antibody levels vs patients' age in the ME/CFS cohort are shown. Each symbol represents an individual subject; linear regression was calculated for all correlations and shown as a black line; *R* and *P*-values are reported in the top right corner. **(c)** Aggregate MFI of all antigens from each patient has been used to calculate the average antibody recognition for each cohort; same approach has been applied to ME/CFS patients.

a

MEDIAN AGE ± IQR	RA	ME/CFS
Whole cohorts (22-84)	51 ± 27	36 ± 12.5
Age matched only (24-55)	40.5 ± 14.5	40 ± 13.5

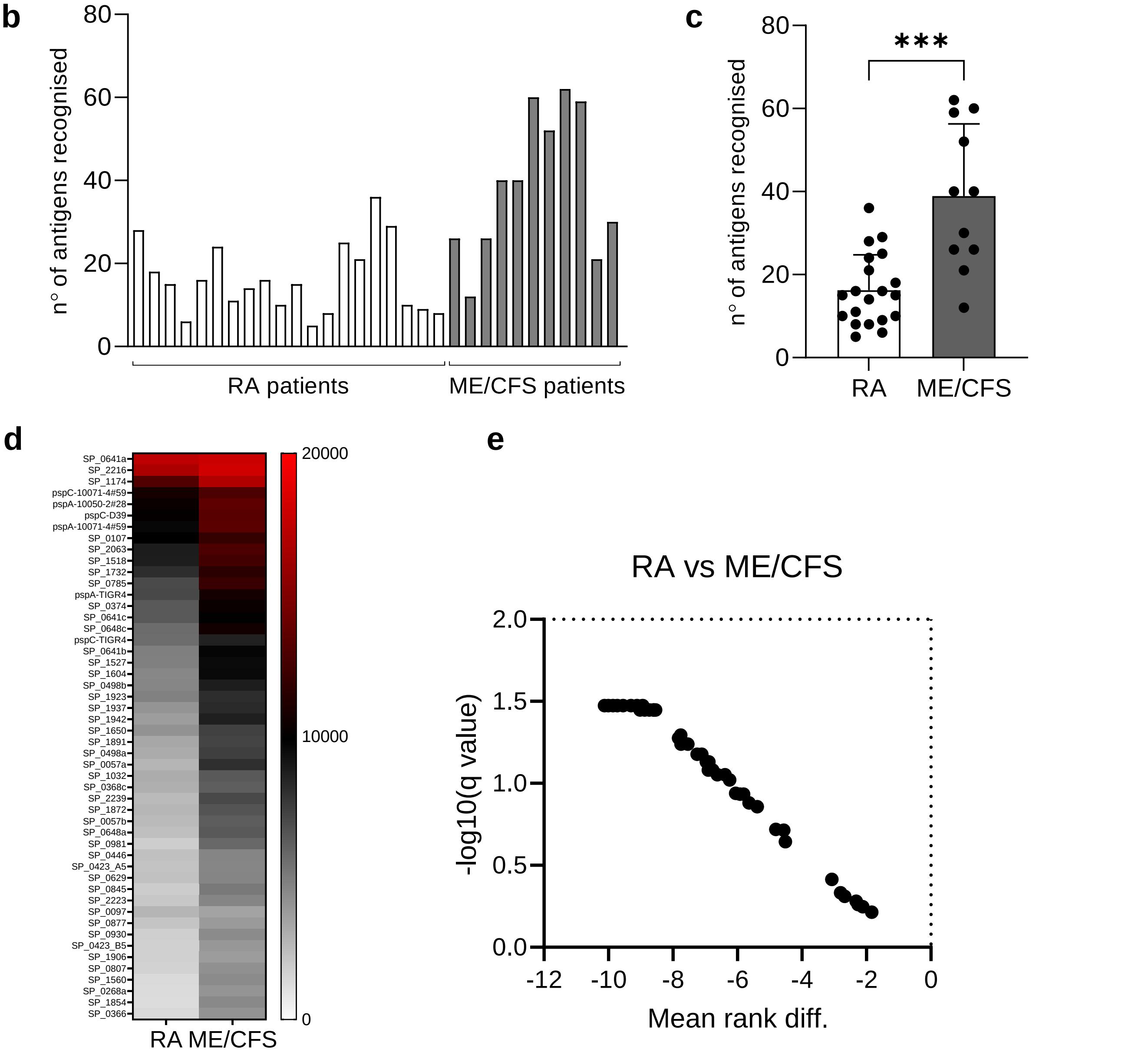

Supplementary figure 3 Analysis of array data considering age-matched cohorts. **(a)** Table showing the median age and IQR of the entire cohorts and the selected age-matched subjects. **(b)** Total number of antigens recognised by age-matched RA and ME/CFS sera. Responses to a specific antigen were considered positive when the MFI measured for the sera was higher than negative control sample (naïve mouse serum) and MFI>3000. **(c)** Overall mean number of antigens recognised for the age-matched RA and ME/CFS groups. Columns indicate mean values and error bars represent standard deviations; the data were analysed using a Mann-Whitney *U*-test (**** = $P < 0.0001$). **(d)** Heatmap of the mean MFI values for the top 50 recognised antigens for the age-matched RA and ME/CFS cohorts ranked from most (top) to lowest (bottom) recognised. Signal strength varies with colour from high (MFI=20000, reds), medium (MFI=10000, black through grey) and low / absent (MFI=0, pale grey to white). **(e)** Volcano plot comparing results for individual antigens between age-matched RA and ME/CFS subjects. Top left quarter represents antigens with higher results in ME/CSF compared to RA subjects (calculated using Mann-Whitney with false discovery rate approach).

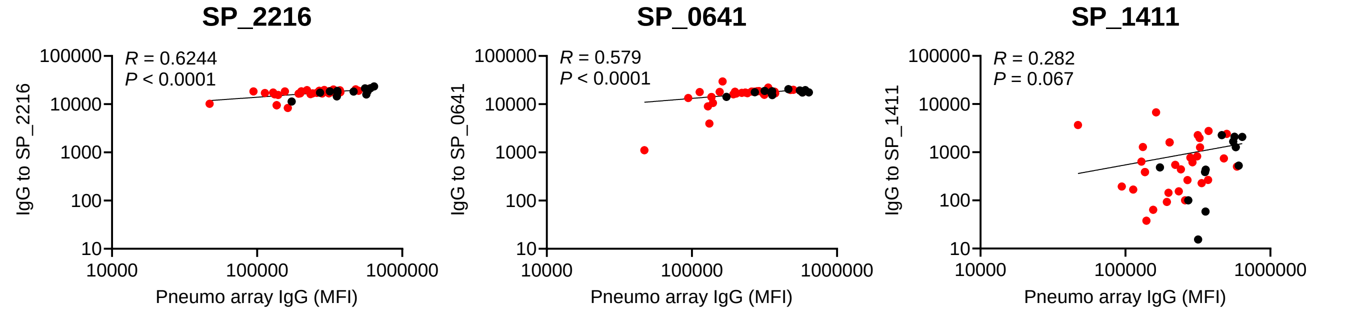

Supplementary figure 4 Anti-pneumococcal array data vs total IgG levels. Correlations between the IgG levels measured using the array for three specific antigens (SP_2216, SP_0641 and SP_1411) and flow cytometry IgG opsonisation data for RA (red symbols) and ME/CFS (black symbols). Each symbol represents an individual subject; linear regression was calculated for all correlations and shown as a black line. *R* and *P*-values are reported in the top right corner.

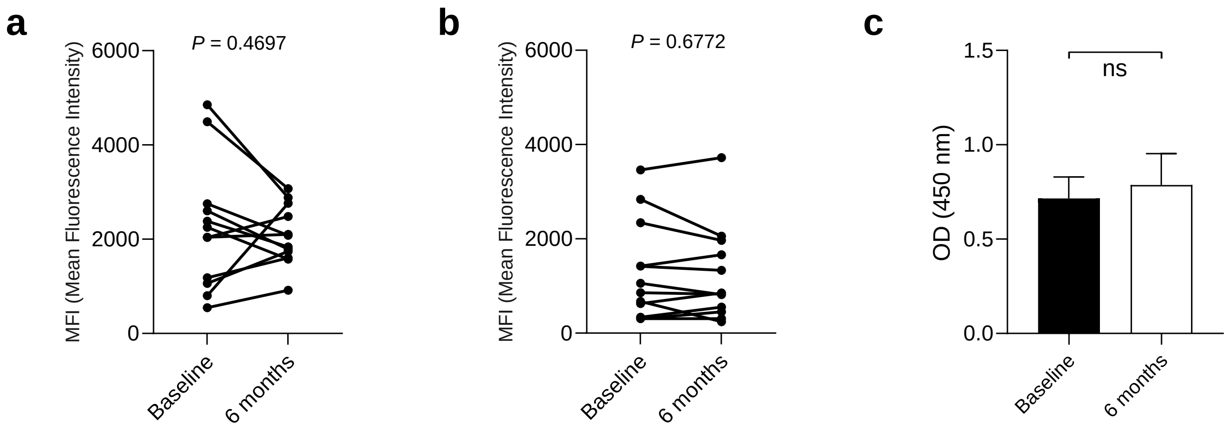

Supplementary figure 5 Level of antibodies against pneumococcus in in ME/CFS patients. Anti-pneumococcus IgG levels were measured in ME/CFS patients sera taken 6 months apart. 6B **(a)** and TIGR4 **(b)** strains were tested for IgG binding. Difference between baseline and post-6 months samples was analysed using Wilcoxon signed-rank test and *P*-values reported in the graphs. **(c)** Anti-pneumococcus IgG were measured by whole cell ELISA against TIGR4 strain at the baseline and after 6 months. Wilcoxon matched-pair signed rank test has been applied to calculate statistical difference between baseline and post-6 months samples (*=*P* < 0.05, ns=not significant).

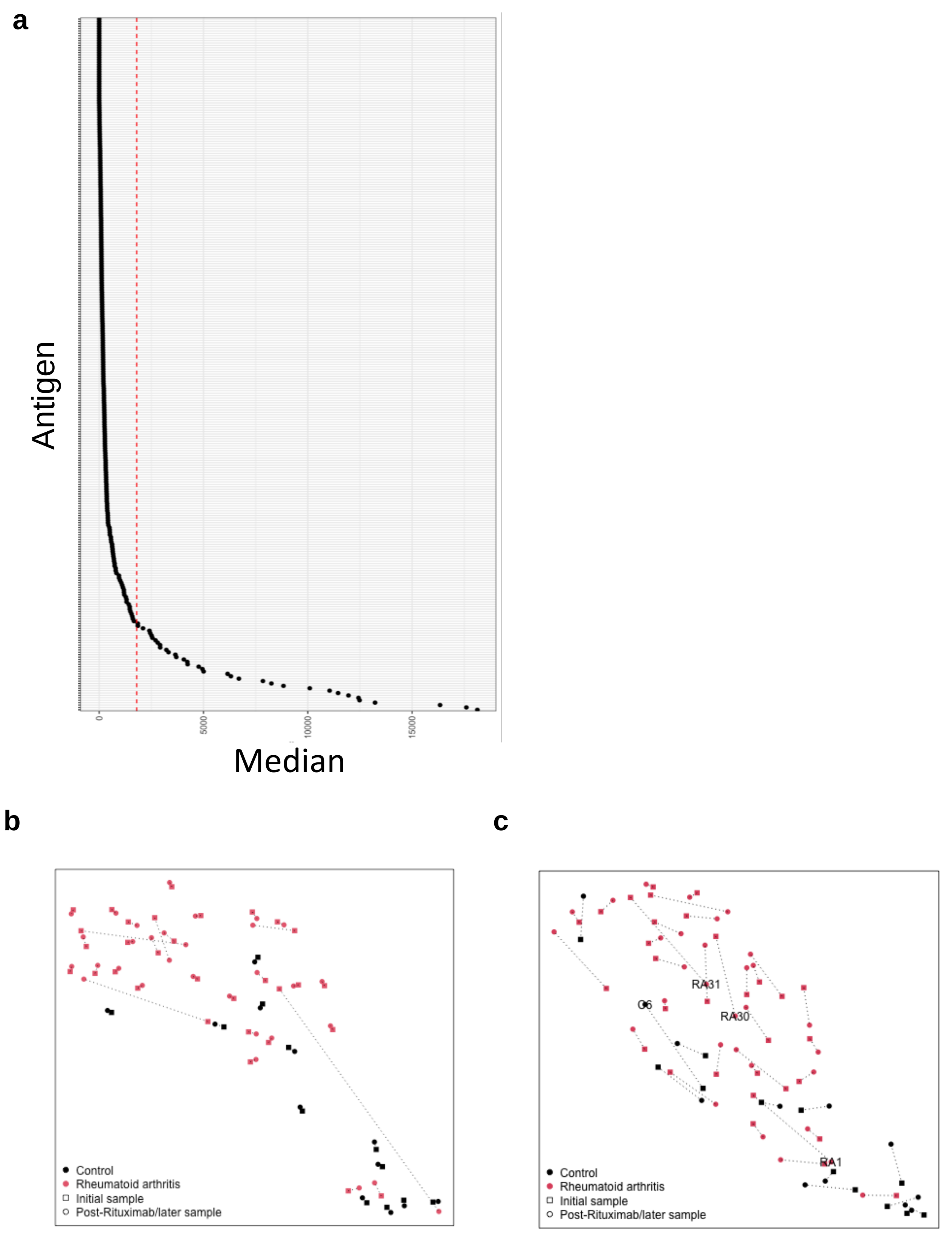

Supplementary figure 6 (a) Plotting the median response to each antigen across all RA individuals it was possible to identify a threshold median response of ~1,800 that separates the top 30 antigens from the rest. **(b)** t-SNE and **(c)** UMAP analysis of protein array data for the top 30 most recognised antigens in pre- vs post-rituximab treatment sera from RA patients (red symbols), and for samples from each ME/CFS (control) subject obtained 6 months apart (black symbols). First sample is reported as a square while the second as a circle.

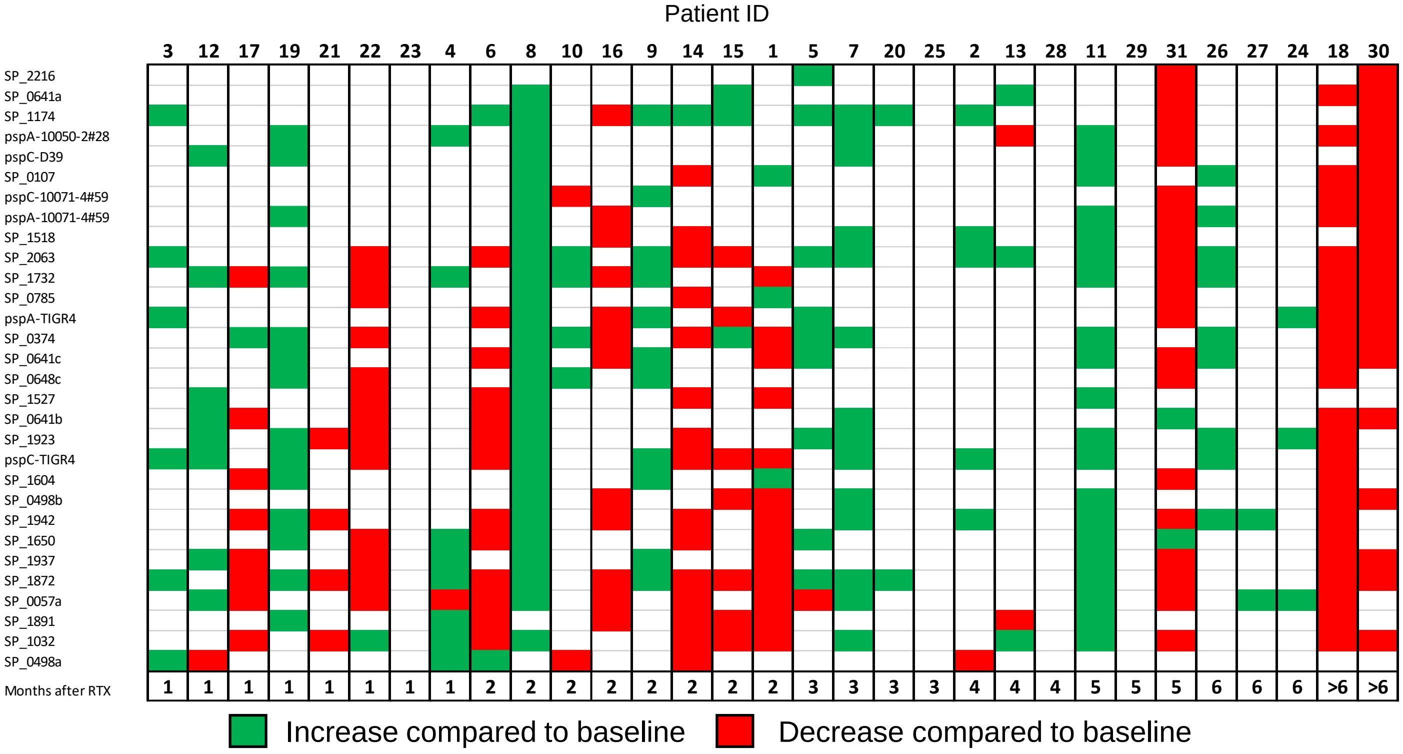

Supplementary figure 7 Variability of anti-protein response after B cell depletion in RA patients. (A) Significant changes in anti-pneumococcal protein response after B cell depletion have been reported for RA patients. Columns identify patients, rows indicate the top 30 ranked antigens in protein array. Coloured cells represent the percentage change outside the standard variability observed in the ME/CFS control cohort (> or < AVG+SD), see supplementary table 1) between pre- and post-depletion samples. Green cells indicate increased IgG levels; red cells indicate decreased IgG levels for each individual antigen. The monthly interval between the pre- and post-rituximab treatment samples for each patient in also reported in the bottom row.
